# Prognostic significance of *NF-κB* expression in non-small cell lung cancer: A meta-analysis

**DOI:** 10.1371/journal.pone.0198223

**Published:** 2018-05-29

**Authors:** Lijun Gu, Zhiyan Wang, Jing Zuo, Hongmei Li, Lin Zha

**Affiliations:** 1 Nanlou Respiratory Diseases Department, Chinese PLA General Hospital, Beijing, China; 2 Nanlou Health Care Department, Chinese PLA General Hospital, Beijing, China; 3 Clinical Center of Spaceport, Chinese PLA General Hospital, Beijing, China; 4 Clinical Center of Spaceport, The 309th Hospital of People's Liberation Army, Beijing, China; Stony Brook University, UNITED STATES

## Abstract

Nuclear factor kappa B (NF-κB), a key nuclear transcription factor, is associated with prognosis in a variety of human cancers. However, the clinical value of NF-κB in non-small cell lung cancer (NSCLC) is still controversial. Therefore, the aim of this meta-analysis was to obtain an accurate evaluation of the relationship between NF-κB expression and survival prognosis of NSCLC patients based on published articles. PubMed, EMBASE and Web of Science databases were systematically searched for potential articles. A total of 1159 patients from 7 eligible studies comparing prognostic significance of NF-κB expression levels in NSCLC were included in our meta-analysis. I^2^ statistic and P value were performed to evaluate heterogeneity. The results of analysis were presented as hazard ratio (HR) or odds ratios (OR) with 95% confidence interval (95% CI). Subgroup analysis based on ethnicity of NSCLC patients and NF-kB cellular localization within cancer cells were conducted to illustrate the potential discrepancy. Significant heterogeneity was considered at I^2^>50% and P<0.05, and random-effects model was used. The combined results indicated that higher NF-κB expression was associated with shorter overall survival (OS) of NSCLC patients (HR = 2.78, 95% CI = 1.51–5.12, P = 0.001). Moreover, NF-κB expression was closely associated with tumor stage (HR = 0.32, 95% CI = 0.18–0.57, P<0.0001), lymph node metastasis (HR = 0.56, 95% CI = 0.38–0.83, P = 0.004) and 5-year OS for NSCLC patients (OR = 1.83, 95% CI = 1.02–3.31, P = 0.04). We conclude that NF-κB expression may be a potential unfavorable prognostic marker for NSCLC patients.

## Introduction

Non-small cell lung cancer (NSCLC) is a major cause of cancer mortality and is one of the most common cancers worldwide [1]. In spite of recent advances in treatment including targeted therapy, adjuvant therapy and surgery, the overall prognosis of NSCLC is grim and the 5-year survival rate is as low as 15% [2, 3]. Therefore, a more favorable prognostic biomarker that contributes to the improvement of survival situation is crucial for the development of therapeutic strategies against NSCLC.

Accumulating evidence has indicated that cancer-related deaths are partially due to activation of inflammatory transcription factors in cancer cells [4–6]. Inflammatory transcriptional factors, including nuclear factor kappa B (NF-κB), regulate the development of malignant tumor through a wide range of physiologic and pathologic processes including cellular senescence, apoptosis, metabolism, stress responses and tumorigenesis [7–11]. NF-κB functions through activating diverse downstream signaling cascades, such as TNFα, BCL-2 and STAT 3 pathways [12–14]. Due to its tumorigenic characteristics, NF-κB may perform as a target for improving living quality for NSCLC patients. Previous studies have shown that NF-κB performs as a tumor promoter in NSCLC. Elevated NF-κB expression levels were detected in NSCLC tissues in contrast with its corresponding normal tissues [15]. Moreover, NF-κB overexpression is associated with cancer cell metastasis and unfavorable prognosis in NSCLC patients [16, 17]. However, the clinical significance of NF-κB on prognostic value is still controversial. Other studies considered NF-κB as a tumor suppressor for NSCLC since it decreased several oncogenes expression and resulted in a better prognostic outcome [18].

In order to explain the discrepancy on the prognostic significance of NF-κB in NSCLC, we conducted a meta-analysis to systematically evaluate the association between the NF-κB expression and NSCLC prognosis.

## Materials and methods

### Publication search

All procedures involved in this meta-analysis were conducted in accordance with the Preferred Reporting Items for Systematic Reviews and Meta-Analyses (PRISMA) Statement.

Literatures published before March 1, 2018 were surveyed thoroughly on PubMed, EMBASE and Web of Science databases. No language limitation and publication restriction were applied. Terms were searched as follows: (“NF-κB” OR “NF-kappa B” OR “nuclear factor kappa B”) AND (“Non-Small-Cell Lung Cancer” OR “Non-Small-Cell Lung Carcinoma” OR “NSCLC” OR “Lung cancer” OR “Lung adenocarcinoma” OR “Lung squamous carcinoma”). The references list of all searched articles was also manually reviewed.

### Article selection

Articles that met the following criteria were eligible for our meta-analysis: investigate the NF-κB expression in NSCLC patients; evaluate the prognostic significance in NSCLC patients with different NF-κB expression; present the values of hazard ratio (HR) with 95% confidence interval (CI) or survival curves. Articles were excluded according to the following criteria: duplicated or overlapped studies; reviews or case reports; animal or database studies; no sufficient original data or unavailable raw data.

### Data extraction

We extracted the following information from qualified studies: names of first author, publication year, country, ethnicity, sample size, values of HR and 95% CI, NF-kB subunit, cellular localization, histology, tumor stage and lymph node metastasis. Two reviewers extracted the data from each of these studies and assessed risk for bias according to the PRISMA recommendations independently.

### Statistical analysis

The pooled HR at a 95% CI was analyzed using Review Manager 5.3 to evaluate the association of NF-κB expression with NSCLC patient survival, and pooled odds ratio (OR) with 95% CI was analyzed to assess the association between NF-κB expression and clinical features. Engauge Digitizer version 4.1 was used to get the values of HRs and 95%CI from survival curves. X^2^-based Q test and I^2^ test were used to evaluate the heterogeneity among studies. Significant heterogeneity was considered at P<0.05 and I^2^>50%, and the random-effects model was utilized. Moreover, a sensitivity analysis was conducted to test the consistency of the combined results by Stata13.0. Additionally, publication bias among selected studies was assessed by Begg’s and Egger’s test using Stata13.0. Statistical significance was considered at p<0.05.

## Results

### Study selection

**Fig 1:**
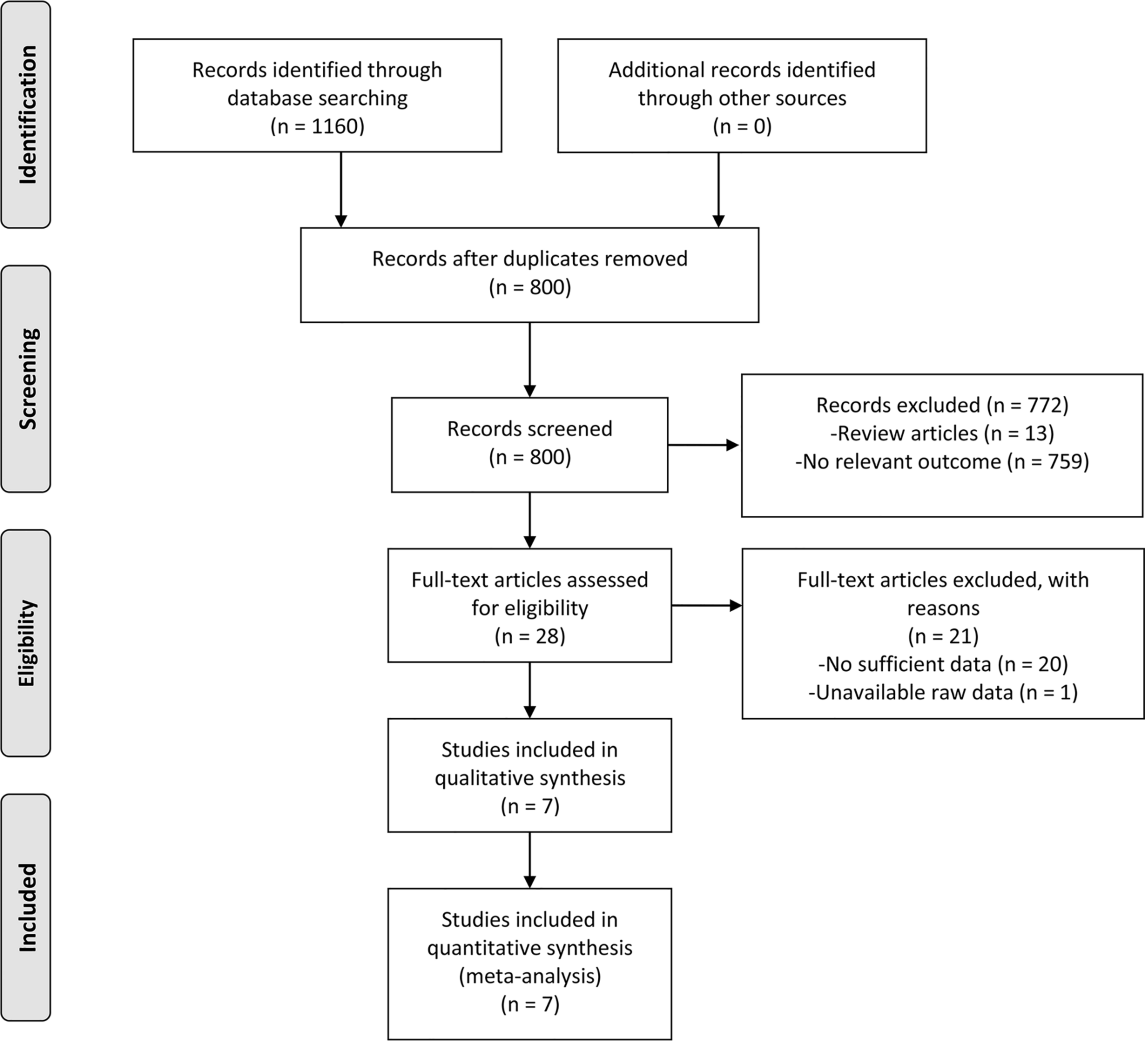
Flow illustration for studies selection process.

A total of 1160 literatures accessed for eligibility were obtained from PubMed, EMBASE and Web of Science databases. During further review, 360 articles were excluded from the analysis due to duplicate publications, 13 studies were deleted due to review articles, 759 articles were excluded due to no relevant outcome, 20 articles removed were due to no sufficient data, 1 articles removed were due to unavailable raw data. Eventually, 7 studies were eligible for our meta-analysis [15–21]. The selection workflow was illustrated in Fig 1.

### Study characteristics

Among the 7 studies, the overall number of cases was 1159 and the sample size ranged from 45 to 345. Five studies were carried out in China, followed by Norway and the USA. Two studies were performed on the Caucasian population, while other studies were on Asians. All studies used immunohistochemistry (IHC) to detect NF-κB expression levels. Three studies explored the relationship between NF-κB expression and histology classification, and four studies detected the association between NF-κB expression and either tumor stage or lymph node metastasis. The main features of the included studies were summarized in Table 1.

**Table 1:** Main characteristics of included studies in the meta-analysis.

First author	Year	Country	Ethnicity	Sample size	HR and 95%CI	NF-kB subunit	Cellular localization	Histology	Tumor stage	LNM
								ADC/SCC	T1T2/T3T4	No/Yes
Jin (15)	2008	China	Asian	88	Reported	P65	Nucleus	23/40	75/13	58/30
Nair (16)	2013	USA	Caucasian	345	Reported	P65	Cytoplasm	NR	NR	NR
Qin (17)	2016	China	Asian	115	Reported	RelB	Cytoplasm	83/32	86/29	48/67
Saad (18)	2008	Norway	Caucasian	335	Reported	P105	Cytoplasm	NR	NR	NR
Yu (19)	2015	China	Asian	115	Reported	P65	Nucleus	NR	103/12	65/50
Zhang (20)	2006	China	Asian	45	Survival Curve	P50	Nucleus	NR	NR	NR
Zhang (21)	2007	China	Asian	116	Survival Curve	P65	Nucleus	31/52	100/16	76/40

HR, hazard ratio; CI, confidence interval; ADC, adenocarcinoma; SCC, squamous cell carcinoma; LNM, lymph node metastasis; NR, not reported.

### Overall analyses

**Fig 2:**
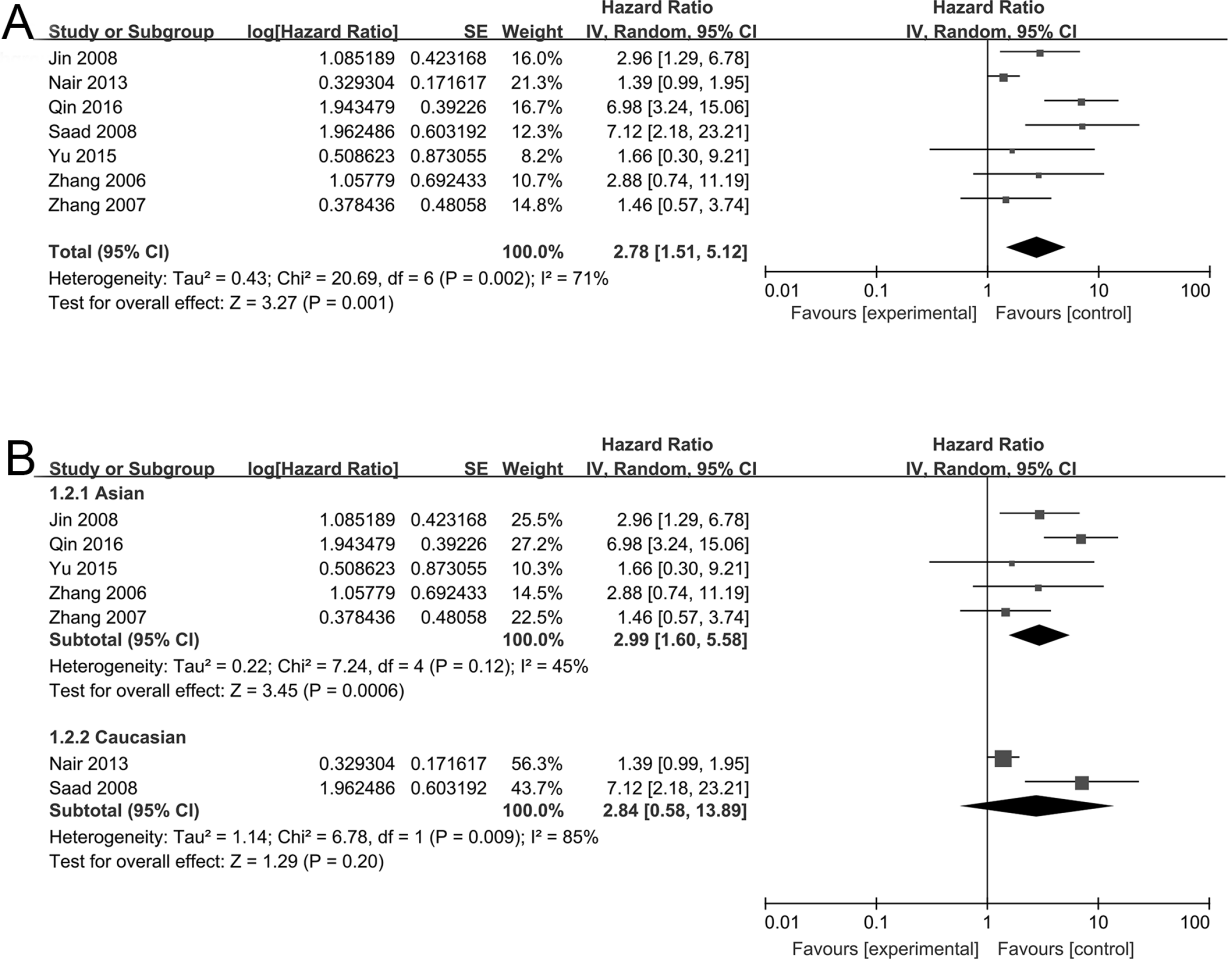
Forest plot for the association between NF-κB and overall survival in NSCLC patients. (A) Overall analysis of all NSCLC patients. (B) Subgroup analysis of Asian and Caucasian NSCLC patients. HR, hazard ratio; CI, confidence interval.

The values of HR and 95% CI from each study were combined to be analyzed (Fig 2). The outcome indicated that higher NF-κB expression was linked to worse overall survival (OS) for NSCLC patients, suggesting a tumor promotive function of NF-κB. (HR = 2.78, 95% CI = 1.51–5.12, P = 0.001). Significant heterogeneity was observed (P = 0.002, I^2^ = 71%), and a random-effects model was used for statistical analysis (Fig 2A). Studies were regrouped for subset analysis based on the ethnicity of NSCLC patients. Outcome indicated that NF-κB expression was significantly correlated with OS in Asian (n = 5, HR = 2.99, 95% CI = 1.60–5.58, P = 0.0006), but not in Caucasian (n = 2, HR = 2.84, 95% CI = 0.58–13.89, P = 0.20) (Fig 2B).

**Fig 3:**
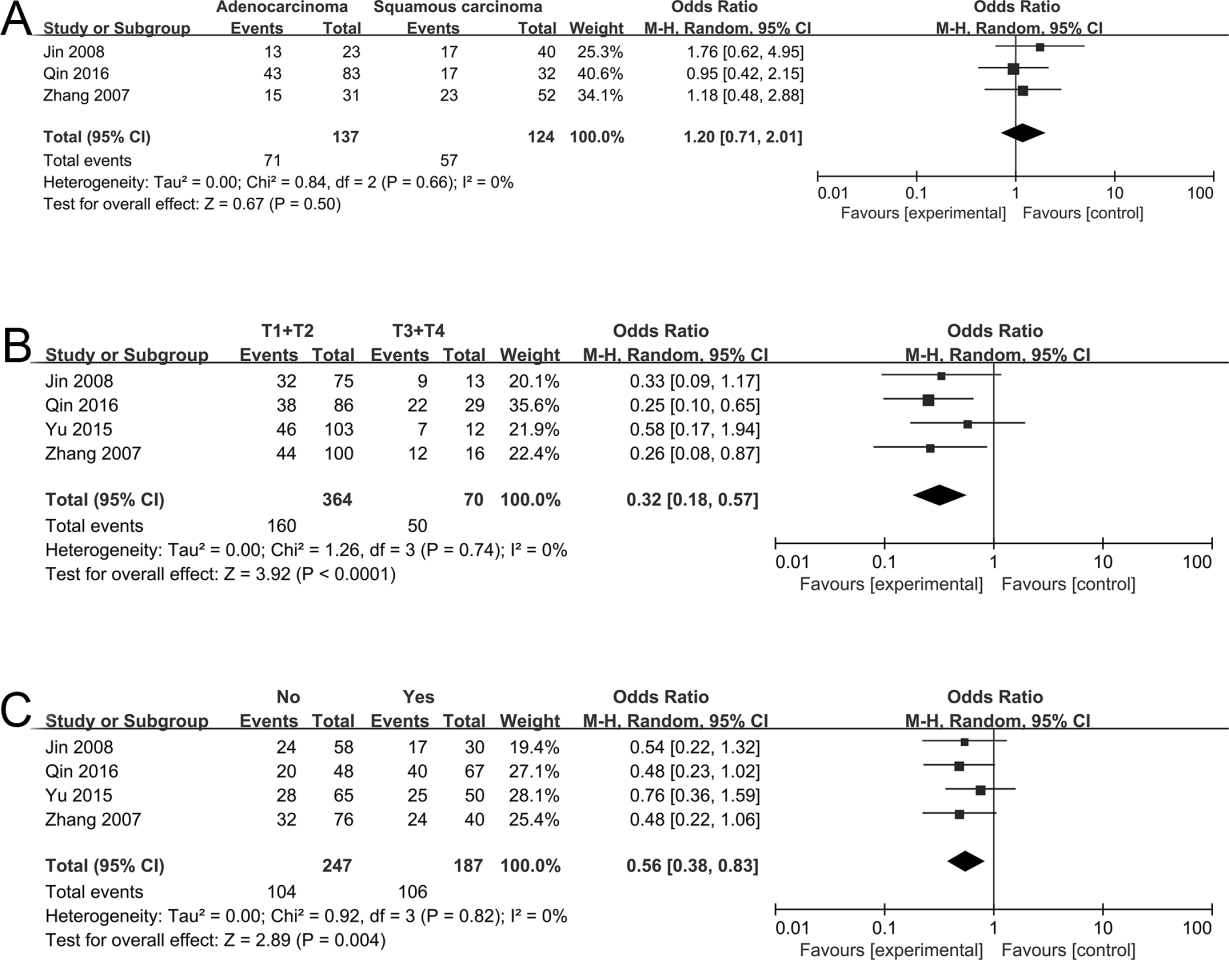
Forest plot for the association of NF-κB with clinicopathological parameters. (A) Patients with adenocarcinoma and squamous cell carcinoma. (B) Patients with tumor stage T1/T2 and T3/T4. (C) Patients with or without lymph node metastasis. OR, Odds ratio; CI, confidence interval.

Three studies explored the relationship between NF-κB and histology, and four studies investigated the correlation between NF-κB and either tumor stage or lymph node metastasis. The pooled OR from 137 lung adenocarcinoma (ADC) and 124 squamous cell carcinoma (SCC) revealed that there was no significant difference of NF-κB expression between ADC and SCC (OR = 1.20, 95% CI = 0.71–2.01, P = 0.50) (Fig 3A). NF-κB expression was frequent in patients with T3/T4 tumor stage rather than T1/T2 (OR = 0.32, 95% CI = 0.18–0.57, P<0.0001) (Fig 3B). Moreover, NF-κB expressed more frequently in patients with lymph node metastasis (OR = 0.56, 95% CI = 0.38–0.83, P = 0.004) (Fig 3C).

**Fig 4:**
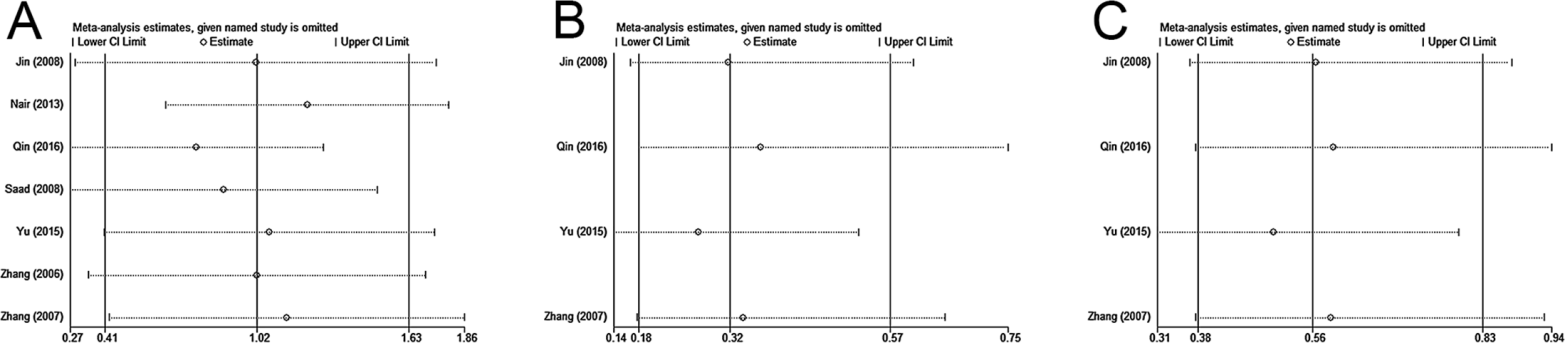
Sensitivity analyses. (A) Overall survival in NSCLC patients. (B) Tumor stage. (C) Lymph node metastasis.

**Fig 5:**
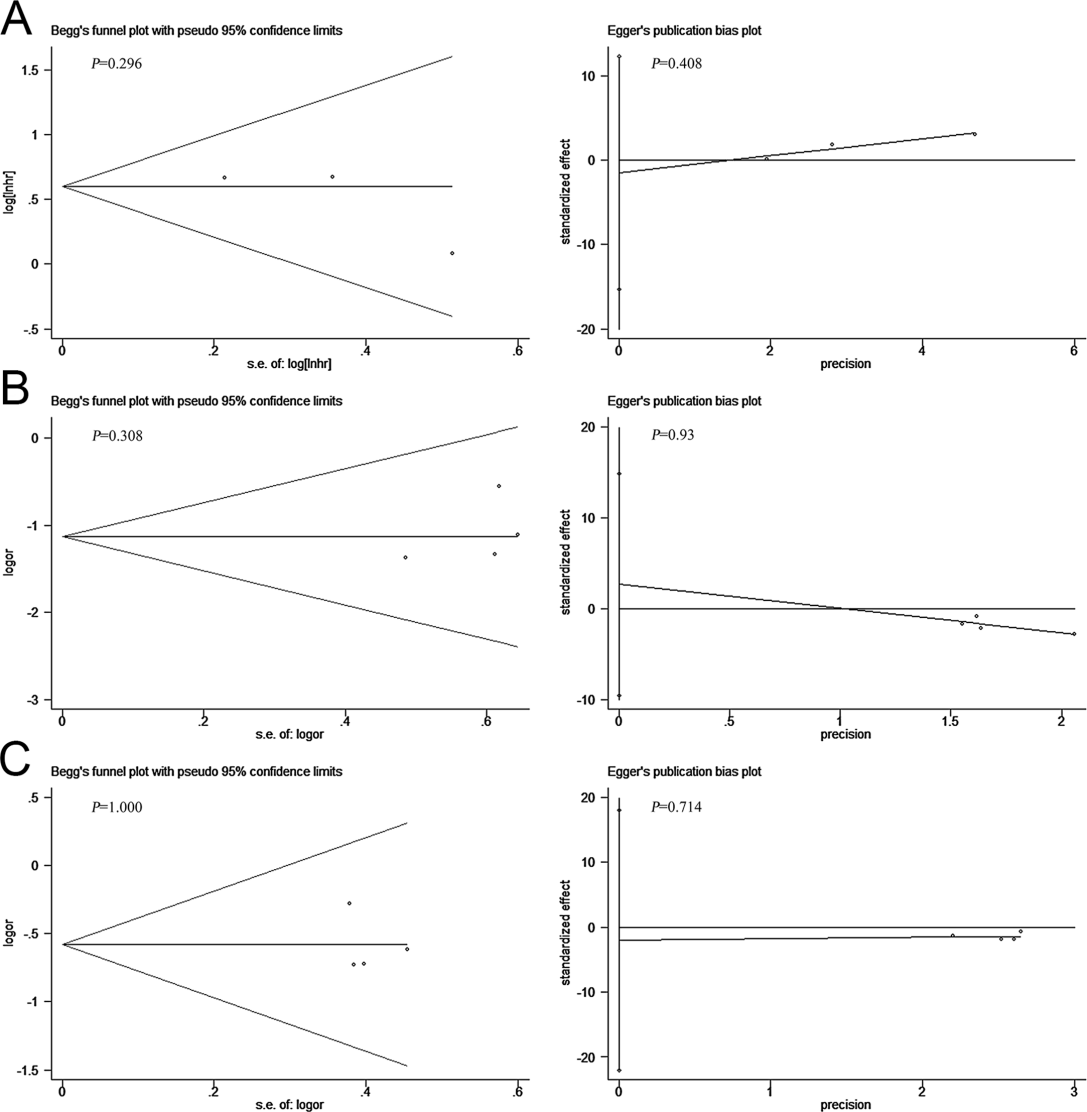
Publication bias estimated by Begg’s and Egger’s test. (A) Overall survival in NSCLC patients. (B) Tumor stage. (C) Lymph node metastasis.

Sensitivity analysis was performed to evaluate the stability of our results. Fig 4 showed that individual study had little substantial impact on the final outcomes, indicating that our results were stable. Publication bias was investigated by both Begg’s and Egger’s test, indicating there was no publication bias in all groups since P>0.05 (Fig 5).

**Fig 6:**
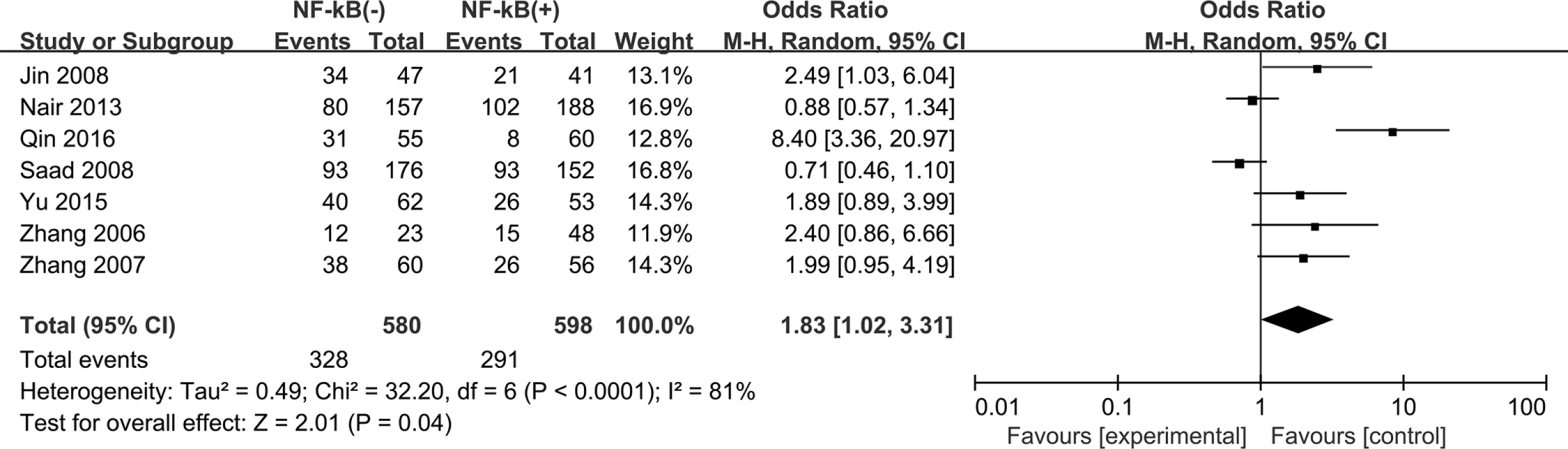
Forest plot for the association between NF-κB expression levels and 5-year overall survival amoug NSCLC patients.

**Fig 7:**
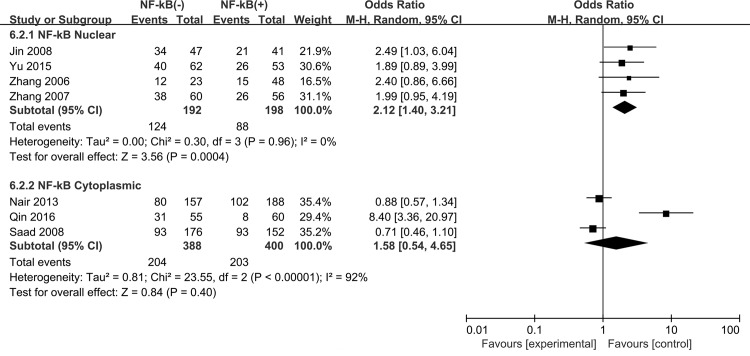
Subgroup analysis of nuclear and cytoplasmic NF-kB expression associated with 5-year overall survival. NF-kB(-), low/negative expression of NF-kB; NF-kB(+), high and moderate/positive expression of NF-kB.

**Fig 8:**
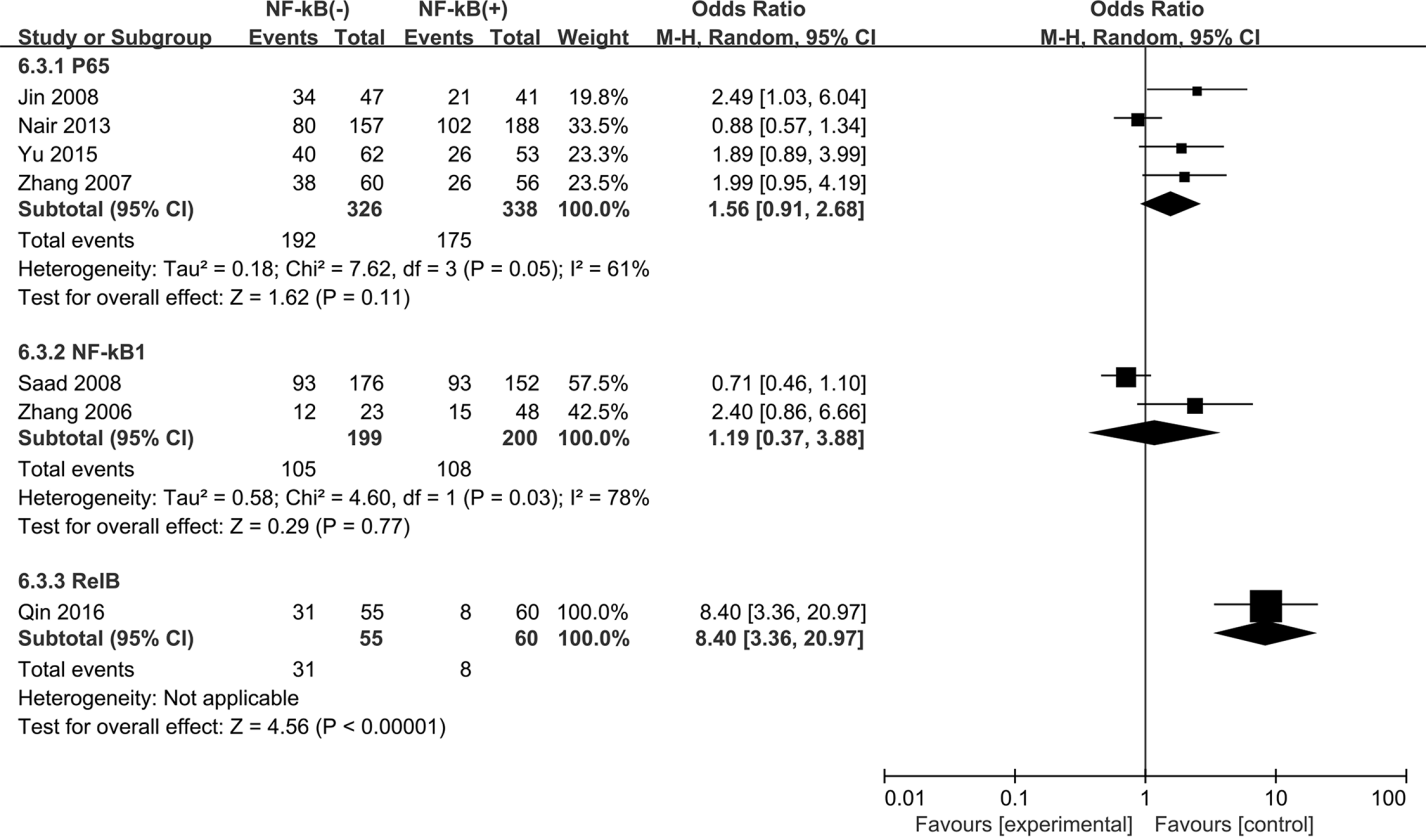
Forest plot for the expression levels of NF-kB family members and 5-year overall survival in NSCLC patients.

We found that high expression of NF-kB correlated with decreased 5-year OS for NSCLC patients (OR = 1.83, 95% CI = 1.02–3.31, P = 0.04) with a significant heterogeneity (P<0.0001, I^2^ = 81%) (Fig 6). Subgroup analysis was carried out based on the NF-kB expression in either nucleus or cytoplasm. We observed that NF-kB expression in nucleus (activation status) was correlated with decreased 5-year OS (OR = 2.12, 95% CI = 1.40–3.21, P = 0.0004), whereas no significant association was observed between cytoplasmic NF-kB expression (inactivation status) and 5-year OS in NSCLC patients (OR = 1.58, 95% CI = 0.54–4.65, P = 0.40) (Fig 7). In addition, the expression levels of NF-kB subunits (P65, NF-kB1 and RelB) were not associated with deceased 5-year OS of NSCLC (P65 OR = 1.56, 95% CI = 0.91–2.68, P = 0.11; NF-kB1 OR = 1.19, 95% CI = 0.37–3.88, P = 0.77; RelB OR = 8.40, 95% CI = 3.36–20.97, P<0.00001) (Fig 8).

## Discussion

It has been documented that NF-κB, an important inflammatory transcriptional factor, performs a pivotal part in various biological processes and has a dual effect on the progression of cancers [22, 23]. Some researchers have identified high expression levels of NF-κB in variety of solid malignancies, such as breast cancer, renal cell carcinoma and oral squamous cell carcinoma [24–26]. Inhibitors targeting at NF-κB expression also inhibited the tumor formation and angiogenesis capacity of lung cancer cells [27]. On the contrary, some studies have shown that NF-κB transactivated the expression of pro-apoptosis genes including Fas and death receptors, and NF-κB performed as a tumor suppressor to facilitate P53-related cancer cell death [28–30]. Therefore, NF-κB may act as different roles in diverse types of carcinoma. As to the prognosis of NSCLC, the relationship of NF-κB expression with survival outcome remains uncertain. Previous studies have reported that NSCLC patients are characterized by enhanced NF-κB expression. However, other studies have demonstrated reduced level of NF-κB expression observed in NSCLC cells. Understanding the prognostic value of NF-κB in NSCLC patients may provide insights for the improvement of clinical outcome. Therefore, our meta-analysis exploring the prognostic role of NF-κB in NSCLC patients is clinically significant.

In this study, we showed that high NF-κB expression as a prognostic predictor is positively associated with poor survival outcome of NSCLC patients. The subgroup analysis unveiled that NF-κB expression was more closely associated with OS in Asian but not in Caucasian, indicating that the heterogeneity may be attributed to different ethnicity of patients. We also investigated the association between NF-κB expression and clinicopathological parameters. We found that NF-κB expressed highly in NSCLC patients with T3/T4 tumor stage and lymph node metastasis, indicating that NF-κB may perform tumorigenesis function in NSCLC. Besides, the expression of NF-kB in nucleus is significantly associated with worse 5-year OS for NSCLC, but not in the cytoplasm, suggesting that the relationship between NF-kB expression and 5-year OS was relevant to subcellular localization of NF-kB. Meanwhile, no association of NF-kB subunits expression and 5-year OS was obtained, revealing that individual expression levels of P65, NF-kB1 and RelB were probably not correlated with 5-year OS for NSCLC patients. More multi-center and well-matched cohort studies will be urgently needed in the future to address the specific function of NF-kB family members on NSCLC prognosis.

The mammalian NF-kB family has subunits including RelA (P65), RelB, NF-kB1 (P50/P105), NF-kB2 (P52/P100) and c-Rel. The pathways of NF-kB activation consist of canonical (or classical) pathway mediated by RelA/P50 or c-Rel/P50 dimers and non-canonical (or alternative) pathway mediated by RelB/P52 dimer [31, 32]. Studies have suggested that canonical pathway generally performs as tumor promoting and anti-apoptotic roles, while non-canonical pathway has tumor suppressing and facilitating apoptosis effects within cancer cells. As to the complexity of NF-kB in NSCLC, further studies are needed to clarify the prognostic value of canonical and non-canonical NF-kB activation pathways in NSCLC patients based on multiple clinical studies in the future. Some literatures have reported paradoxical prognosis of NF-kB in different cohort of NSCLC patients. This discrepancy can be explained by the fact that NF-kB functions under regulations of diverse upstream driving mutations, including oncogene gain of function and/or loss of tumor suppressors, which ascertains its effects on regulating various downstream targets to trigger either tumor promoting or tumor suppressing function within cancer cells.

Although immunohistochemical method has been extensively used for decades, IHC performed by different laboratories has potential flaws for evaluating candidate prognostic biomarkers due to utilizing different antibodies and/or immunohistochemical protocols [33–36]. As to the enrolled articles analyzed in this meta-analysis, all antibodies used were commercially available for IHC assay, and IHC protocols were performed based on standard peroxidase-based staining methods, including streptavidin-peroxidase method and avidin-biotinylated peroxidase complex method. Hence, the immunohistochemical results reported in these included studies are reproducible. Our study, however, found variable factors during IHC staining procedures in these enrolled studies, such as differences of antibodies used, incubation conditions and thresholds for positive immunostaining, which might bring in misleading information. In view of the above shortcomings, we suggest that IHC, at least in some cases, might not be an optimal method for evaluating candidate prognostic biomarkers in cancer tissues. Thus, more standardized protocols such as mining transcriptomic datasets from cancer tissues are vital for improving results reproducibility. And further meta-analysis is warranted to compare IHC to other genetic analysis.

Several limitations of this study might be pointed out. First, the sample size of cases in the selected studies was relatively limited in a range from 45 to 345, which could have resulted in inaccuracy in this meta-analysis. Second, the staining intensity cut-off values of NF-κB expression analyzed by IHC, a semi-quantitative evaluation method, were observer-dependent in these studies, which may affect the heterogeneity in our analyses. Third, the selected studies in our analyses were conducted mainly in Asian countries; little is known about the association of NF-κB expression with NSCLC in other countries. So more multi-center studies are warranted to verify the role of NF-κB in NSCLC prognosis.

In conclusion, we first combine the results of multiple clinical studies to reveal an association of NF-κB expression with OS in patients with NSCLC. The discovery of NF-κB as a predictor for the poor prognosis of NSCLC patients in our study might be important clinically.

## Supporting information

S1 Table PRISMA checklist. (DOC)
